# Probiotics Modulate Host Immune Response and Interact with the Gut Microbiota: Shaping Their Composition and Mediating Antibiotic Resistance

**DOI:** 10.3390/ijms241813783

**Published:** 2023-09-07

**Authors:** Walaa K. Mousa, Sara Mousa, Rose Ghemrawi, Dana Obaid, Muhammad Sarfraz, Fadia Chehadeh, Shannon Husband

**Affiliations:** 1 College of Pharmacy, Al Ain University, Abu Dhabi P.O. Box 64141, United Arab Emirates; 202010763@aau.ac.ae (S.M.); rose.ghemrawi@aau.ac.ae (R.G.); dana.obaid@aau.ac.ae (D.O.); muhammad.sarfraz@aau.ac.ae (M.S.); 2 AAU Health and Biomedical Research Center, Al Ain University, Abu Dhabi P.O. Box 112612, United Arab Emirates; 3 College of Pharmacy, Mansoura University, Mansoura 35516, Egypt; 4 Anschutz Medical Campus, Colorado School of Public Health, University of Colorado, Aurora, CO 173364, USA; fadia.chehadeh@cuanschutz.edu; 5 Department of Biology, Whitman College, Walla Walla, WA 99362, USA; sghusband@icloud.com

**Keywords:** probiotics, antibiotics, resistance, microbiome, inflammation, immune reactions

## Abstract

The consortium of microbes inhabiting the human body, together with their encoded genes and secreted metabolites, is referred to as the “human microbiome.” Several studies have established a link between the composition of the microbiome and its impact on human health. This impact spans local gastrointestinal inflammation to systemic autoimmune disorders and neurodegenerative diseases such as Alzheimer’s and Autism. Some of these links have been validated by rigorous experiments that identify specific strains as mediators or drivers of a particular condition. Consequently, the development of probiotics to compensate for a missing beneficial microbe(s) has advanced and become popular, especially in the treatment of irritable bowel diseases and to restore disrupted gut flora after antibiotic administration. The widespread use of probiotics is often advocated as a natural ecological therapy. However, this perception is not always accurate, as there is a potential for unexpected interactions when administering live microbial cultures. Here, we designed this research to explore the intricate interactions among probiotics, the host, and microbes through a series of experiments. Our objectives included assessing their immunomodulatory effects, response to oral medications, impact on microbial population dynamics, and mediation of antibiotic resistance. To achieve these goals, we employed diverse experimental protocols, including cell-based enzyme -linked immunosorbent assay (ELISA), antibiotic susceptibility testing, antimicrobial activity assays, computational prediction of probiotic genes responsible for antibiotic resistance, polymerase chain reaction (PCR)-based validation of predicted genes, and survival assays of probiotics in the presence of selected oral medications. Our findings highlight that more than half of the tested probiotics trigger an inflammatory response in the Caco-2 cell line, are influenced by oral medications, exhibit antibacterial activity, and possess genes encoding antimicrobial resistance. These results underscore the necessity for a reevaluation of probiotic usage and emphasize the importance of establishing regulations to govern probiotic testing, approval, and administration.

## 1. Introduction

Trillions of diverse microbial species inhabit our body, collectively referred to as the human microbiome [1]. The composition of the microbiome is shaped by multiple factors, starting with the mode of delivery at birth and varying with diet, lifestyle, aging, diseases, and drugs [2]. While a diverse and balanced composition mediates health benefits, a dysbiosis shift drives diseases ranging from inflammation and metabolic dysregulation to neurological disorders and depression [3]. An appealing strategy for restoring health status is the use of probiotics, which are defined as “live microorganisms that, when administered in adequate amounts, confer a health benefit on the host [4].

In theory, probiotics restore balanced gut microbiota and contribute health benefits while being generally safe [5,6]. This approach is becoming popular, especially in the treatment of recurrent infections, to compensate for the depletion of the microbiota after administration of broad-spectrum antibiotics [7] or to alleviate symptoms of several pathological conditions such as irritable bowel diseases [8,9,10,11], obesity [12,13], infectious diseases [14], colorectal cancer [15], insomnia and depression [16], and obsessive-compulsive disorder [17]. The most used probiotics are members of the *Lactobacillus* and *Bifidobacterium* genera [18]. For example, the use of the probiotic strain *L. rhamnosus* SHA113 in vitro and in vivo shows some promise in managing multi-drug-resistant *S. aureus* infections [19]. *Bifidobacterium* probiotics are used as an adjuvant therapy in the treatment of *C. difficile* infection [20]. Multiple probiotic strains show effectiveness in decreasing the inflammation associated with IBD [21]. In addition, probiotic supplements are utilized in various conditions, even those that are not directly related to GIT, such as depression [22], Parkinsons disease, and Rheumatoid Arthritis [23]. The probiotic bacterial species *L. rhamnosus* can also increase glutamine, glutathione, and N-acetylaspartylglutamate to ameliorate symptoms of depression [24]. Additionally, *L. acidophilus* and *L. salivarius* provide neuroprotective and anti-inflammatory effects in patients suffering from Parkinson’s disease [25]. Certain strains from the genus *Bifidobacterium* show effects in regulating the luteinizing hormone in polycystic ovary syndrome [26].

In contrast to conventional therapeutics, the use of probiotics has been widely promoted as an ecological natural therapy with no to minimal side effects [6]. However, probiotics are live microbial strains with an average of 3500 genes that encode enzymes and chemicals that could interfere with the host and microbial community [27]. The most significant interactions that have been observed include (1) regulation of gene expression in the host or other microbial species by modulating miRNA; (2) production of chemicals such as short-chain fatty acids (SCFAs), bile acid derivatives, antimicrobial peptides, and lipopolysaccharides that can affect host cells or affect the population abundance of other gut microbes; and (3) encoding resistance genes transferable to other microbes and thus mediating the spread of antibiotic resistance [28,29,30,31]. Long-term use of probiotics, coupled with exposure to antibiotics and the resulting selection pressure, poses a potential risk for the emergence of multi-drug-resistant strains [32]. These genes could be transferred to opportunistic pathogens and commensal residents within the gut microbiota community [33]. For example, some *Lactobacillus* species have been confirmed to transfer antibiotic resistance genes to *E. coli* [34]. Previous research identified AMR genes associated with gene mobilizing elements such as plasmids or phages in probiotic strains [35] which could be employed to mobilize these genes via horizontal gene transfer [31]. A dramatic example of the unexpected interaction of probiotics is *Escherichia coli* Nissle (1917). The strain is one of the first commercially available probiotics and was later identified to contain a gene cluster implicated in mammalian colorectal cancer [36]. Other data shows that the use of probiotics may worsen the original conditions; for example, a meta-analysis of randomized controlled trials revealed that probiotics worsen the abdominal pain associated with inflammatory bowel disease [37,38]. Some other side effects reported with the use of probiotics included sepsis [39,40,41], bacteremia [42], endocarditis [43,44], allergic rhinitis [45], and sensitization [46].

Currently, there is an intriguing interest in identifying new probiotics with applications to varied diseases from obesity and neurological disorders [12,17]. However, given the potential interactions, the indiscriminative and unregulated use of probiotics might pose potential health risks such as the transfer of antibiotic resistance genes. Previous reports underscore the importance of enhanced regulation and revised safety considerations for probiotic use [47]. Here, we aimed to investigate different forms of interactions and activities of selected commercial probiotics with host and microbes including immunomodulatory activity in human cell lines, antibiotic-like activity against other microbes, antibiotic resistance potential, and interaction with oral medications.

## 2. Results

In this study, our primary objective was to investigate potential interactions between probiotics, the host, and other gut microbes. To achieve this, we conducted a series of experiments aimed at assessing their impact on the immune response in human cell lines, their compatibility with oral medications, their antimicrobial properties, and their role in mediating antibiotic resistance. As outlined below, our findings revealed that probiotics exhibit a selective antimicrobial-like effect that could potentially influence the structure of the gut microbiota. Additionally, they carry multiple transmissible antibiotic resistance genes.

**Figure 1:**
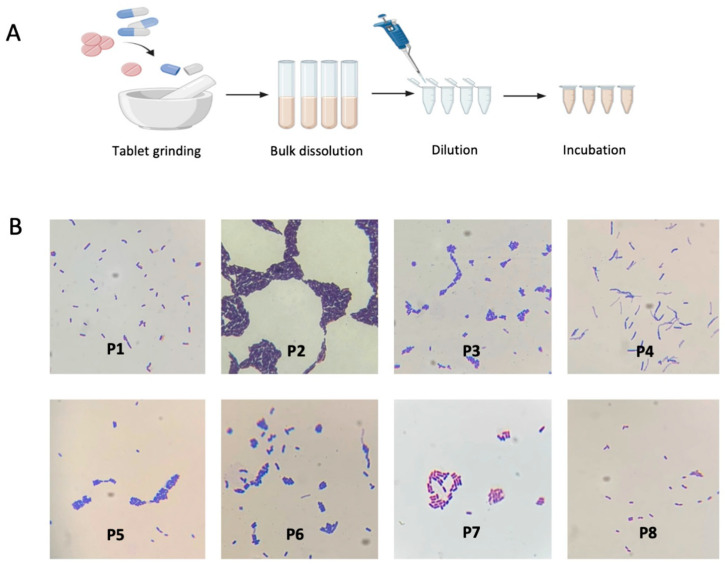
Recovery of probiotic strains from the selected commercial probiotics. (**A**). diagrammatic sketch illustrates the process of reviving the strains from the dosage form by suspension, dilution, and culturing on selective media. (**B**). Microscopy images of some revived strains stained with Gram stain and visualized using a compound light microscope with 100× magnification.

We selected eight commercial supplements labeled as probiotics (P1–P8) with varying microbial compositions and intended uses (Supplementary Excel File S1). Following a standard protocol (Figure 1), we successfully revived the corresponding bacterial strains from these probiotic formulations. We employed a combination of morphological, biochemical, and molecular techniques to assess the recovery rate of distinct bacterial isolates from each probiotic mixture. In each probiotic, we were able to recover at least 60 to 100% of the strains (Supplementary Excel File S1). Although the recovery rate tended to decrease as the number of strains in the probiotic mixture increased, the lowest recovery rate was observed for *Bacillus subtilis* and *Streptococcus thermophilus*. Subsequently, we proceeded to assess the potential interactions of the revived probiotics, as described in more detail below.

### 2.1. Immunomodulatory Activity of the Probiotics

**Figure 2:**
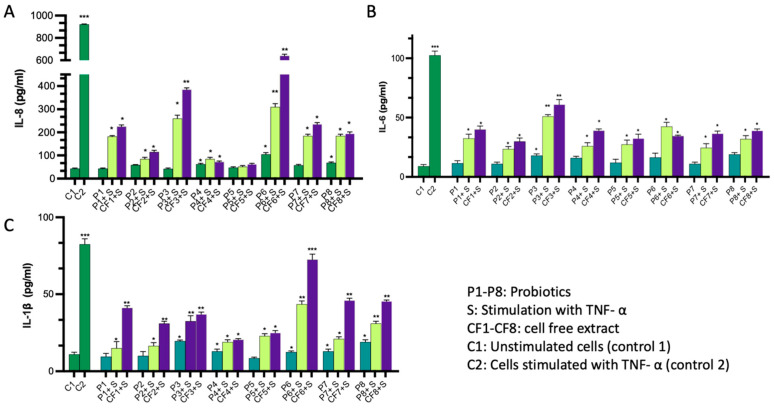
Probiotics exhibit immunomodulatory effects on Caco-2 cell lines. (**A**–**C**) graphs showing the effect of each probiotic (P) or the corresponding cell-free extract (CF) on Caco-2 cells with and without stimulation (S). All measurements were conducted in triplicates and presented as mean and SDs. Error bars represent the SD of the mean. The *, **, and *** indicate that the results are significantly different from the control at *p* < 0.05, *p* < 0.01, and *p* < 0.001, respectively.

To evaluate the immunomodulatory effects of commercial probiotics and their cell-free extracts, we conducted an in vitro assay using Caco-2 cell lines. Briefly, we incubated the probiotics or their cell-free extracts with Caco-2 monolayers, both with and without TNF-α as an inducer, to increase baseline secretion of cytokines. Subsequently, we quantified the levels of secreted cytokines in the culture supernatant using ELISA kits. Our results indicate that certain probiotics led to a statistically significant increase in measured cytokines compared to untreated cells, with a significance threshold of *p* < 0.05 (Figure 2A–C and Supplementary Excel File S2). For example, probiotics P3, P4, P6, and P8 exhibited a change in IL-6 levels, with up to a two-fold increase compared to untreated cells. On average, their values were 18, 16, 17, and 19 pg/mL, respectively, compared to 9 pg/mL in the untreated cells. Similarly, the secretion of IL-8 significantly increased with probiotics P2, P4, P6, P7, and P8, with average values of 59, 62, 105, 57.5, and 67.5 pg/mL, respectively, compared to 43.5 pg/mL in untreated cells. Additionally, the secretion of IL-1β increased by more than 70% with P3 and P8 compared to untreated cells, with average values of 19 and 19.5 pg/mL, respectively, compared to 11 pg/mL in untreated cells. Cell-free extracts demonstrated a similar pattern to the live probiotics, albeit with less pronounced reductions in cytokine release compared to their corresponding live strains. It is crucial to note that these findings are applicable exclusively to in vitro cell line conditions, and further investigations are necessary to validate their relevance in vivo.

### 2.2. Probiotics Exhibit Antimicrobial Activity against a Broad Range of Human-Associated Microbes

**Figure 3:**
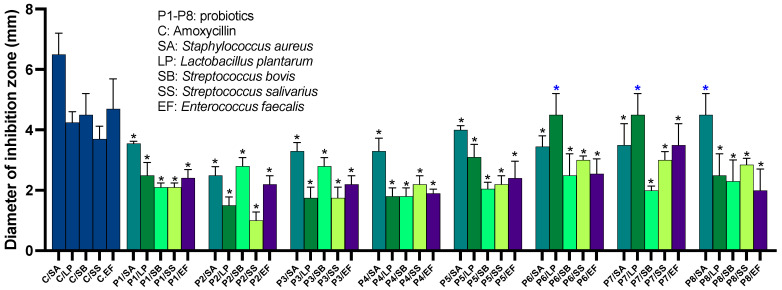
The inhibitory effect of the probiotics on multiple human-associated microbes. A graphical representation showing the diameter of inhibition zones in five different microbes resulted from treatment with antibiotics and probiotics as indicated. All measurements were conducted in triplicate and presented as mean and SDs. Error bars represent the SD of the mean. The * indicates that the results are significantly different from the control at *p*< 0.05. Blue * represents the most potent activity observed.

To assess the antimicrobial potential of the probiotics, we tested their activity against ten microbial species, representatives of the human-associated microbes. These species comprised *Lactiplantibacillus plantarum* subsp. plantarum, *Lacticaseibacillus casei*, *L. rhamnosus*, *Staphylococcus aureus*, *Streptococcus bovis*, *S. salivarius*, *Enterococcus faecalis*, *E. coli*, and *Pseudomonas aeruginosa*, as well as antifungal activity against *Candida albicans* and *Saccharomyces cerevisiae*. Antibiotic controls were included, consisting of amoxicillin, gentamycin, vancomycin, and chloramphenicol. Our findings reveal that all tested probiotics exhibited statistically significant growth inhibition against at least five of the tested microbial species when compared to the control (*p* < 0.05). Notably, *S. aureus* and *L. plantarum* were the most susceptible species to probiotic inhibition (see Figure 3 and Supplementary Excel File S2). Overall, P7 demonstrated the broadest spectrum of activity, followed by P6 and P5, whereas P2 and P3 exhibited the least inhibition overall. Specifically, P6, P7, and P8 inhibited S. aureus with inhibition zone diameters of 4 mm, 4.5 mm, 3.5 mm, and 4.5 mm, respectively, compared to the 6.5 mm inhibition zone caused by amoxicillin. *L. plantarum* was mainly inhibited by P5, P6, P7, and P8, resulting in an inhibition zone of 4.5 mm, compared to the 4.25 mm inhibition zone caused by amoxicillin. P6, P7, and P8 inhibited *S. salivarius*, with inhibition zone diameters of 3 mm, 3 mm, and 2 mm, respectively, compared to the 3.7 mm inhibition zone caused by amoxicillin. For S. bovis, P2 and P3 were effective, resulting in inhibition zones of 2.5 mm, compared to the 4.5 mm inhibition zone caused by the control. The most significant inhibition was observed with P7 against E. faecalis, resulting in a 3.7 mm inhibition zone compared to the 4.7 mm inhibition zone caused by amoxicillin. However, *L. rhamnosus* and *L. casei* showed lower susceptibility to all probiotics compared to other tested strains, with inhibition zone diameters ranging from zero to 0.5 mm. Of note, none of the probiotics significantly inhibited the growth of *E. coli* and *P. aeruginosa*, except for P4, which resulted in clear zones of inhibition with diameters of 0.8 mm and 1.1 mm, respectively. However, none of the probiotics exhibited antifungal activity against the tested fungal species.

#### The Inhibitory Activity of P4 against Gram-Negative Pathogens Is Traceable to a Single Strain from the Probiotic Mixture

Since P4 was the only probiotic that showed activity against Gram-negative microbes, we attempted to identify the isolate that mediates this activity for further studies. We purified isolates based on colony morphology when they grew on different media under various growth conditions. We generated 12 isolates and tested them for their antimicrobial activity against *P. aeruginosa*. Three isolates showed inhibitory activity with varied zones of inhibition (1.5, 1,5, and 1.6 mm against *P. aeruginosa* and 1.2, 1.3, and 1.4 mm against *E. coli*). These isolates were further identified based on the analysis of their 16S rRNA genes by sequencing after PCR amplification (Supplementary Excel File S1). The most active isolates were identified as *Bifidobacterium infantis* with a sequence identity of 98.67% and *Lactobacillus delbrueckii* subsp. lactis (two identical isolates) with a sequence identity of 99%. Further, we confirmed that the cell-free extracts of these two strains maintain similar antimicrobial activity against *P. aeruginosa* and *E. coli* (zones of inhibition equal 0.9 and 1.1 mm, respectively, at a concentration of 10 μg/mL. The results suggest that these isolates might secrete active antimicrobial molecules.

### 2.3. Probiotics Possess Antibiotic Resistance Genes

**Figure 4:**
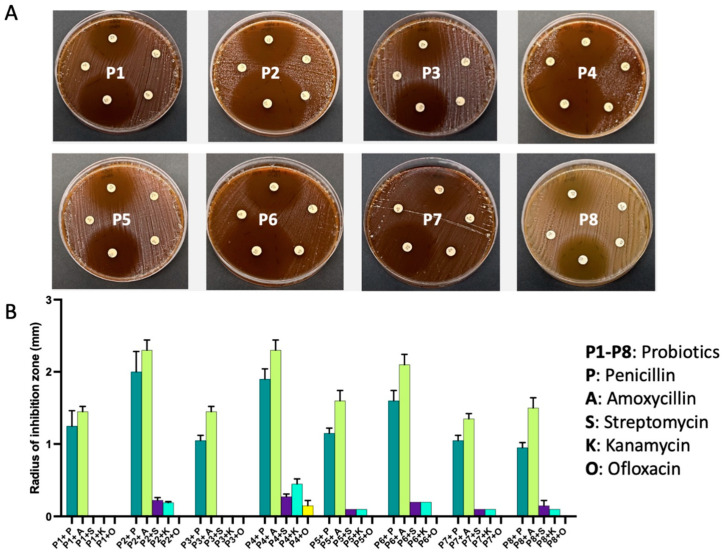
Commercial probiotics exhibit resistance against clinically used antibiotics. (**A**). Images of agar disc diffusion assay showing inhibition zones observed with commercial antibiotics on each probiotic. (**B**). graphical representation of the diameter of inhibition zones with each antibiotic. All measurements were conducted in triplicate and presented as mean and SDs.

To assess if probiotics could medicate against antimicrobial resistance (AMR), we conducted a computational assessment of genes involved in AMR. This involved searching the Comprehensive Antibiotic Resistance Database (CARD) using genomes from 15 bacterial strains that compose these commercial probiotics. The results revealed that every surveyed genome contained AMR gene families, encompassing genes responsible for aminoglycoside antibiotic resistance, tetracycline resistance, and genes inactivating β-Lactams or altering their target (Supplementary Excel File S3). These findings were consistent with earlier studies identifying aminoglycoside-modifying enzymes and tetracycline resistance genes in the *Lactobacillus* genus [48,49]. To validate these computational results, we conducted the following two experiments:

First, we performed an antibiotic susceptibility test to assess whether the selected probiotics exhibited antibiotic resistance. This involved testing each isolate from the probiotics against a panel of five widely used antibiotics, including penicillin G, ampicillin, streptomycin, kanamycin, and ofloxacin. While all eight probiotics displayed sensitivity to penicillin G and ampicillin, they exhibited varying levels of inhibition. Of note, seven of the tested probiotics (P1–P3, P5–P8) demonstrated resistance to ofloxacin without any inhibition. P1 and P3 also displayed resistance to streptomycin and kanamycin (Figure 4).

Second, we selected six candidate resistance genes and performed PCR amplification on distinct strains isolated from the probiotics (see Supplementary Excel File S1). The results confirmed the presence of genes encoding resistance to tetracyclines, specifically *tet*M and *tet*L, in isolates from P3/P6 and P7/P8, respectively (Supplementary Excel File S1). We also detected the presence of *par*C in isolates from P1–P6, though we were unable to amplify other predicted genes using the designed primers. It is important to note that tetracycline resistance genes are predominantly reported in probiotic strains. *Tet*M encodes ribosomal protection proteins, *tet*L encodes efflux pumps, and *par*C is a component of the topoisomerase enzyme crucial for DNA transcription. Mutations in *par*C prevent fluoroquinolone antibiotics from inhibiting DNA synthesis, resulting in resistance, particularly to ciprofloxacin.

### 2.4. Synergistic and Antagonistic Effect of Co-Administered Drugs on Probiotics

**Figure 5:**
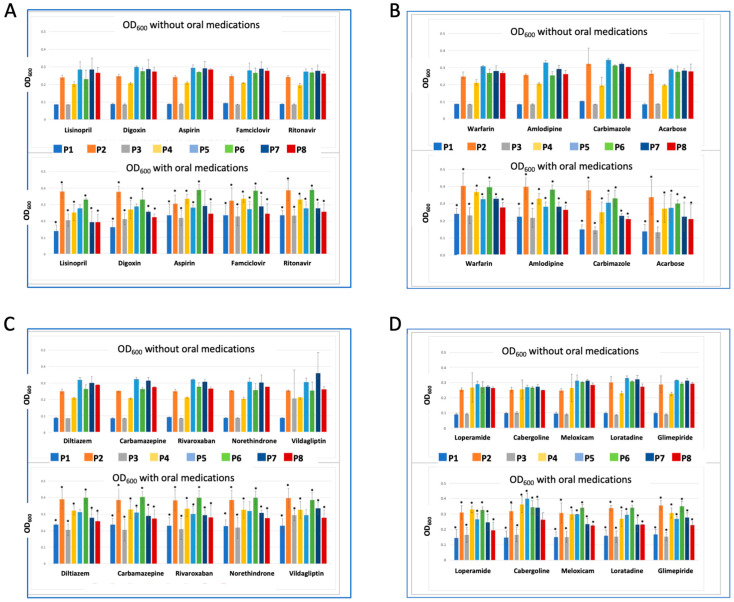
The interaction between some commonly used drugs and microbes. (**A**–**D**) graphs illustrate the OD_600_ of probiotic cultures with and without coincubation with each drug. All measurements were conducted in duplicate and presented as mean and SDs. The * indicates that the results are significantly different from the control at *p* < 0.05.

To assess the effect of co-administering oral medications with probiotics, we conducted a co-incubation assay involving 19 commonly used drugs. In this assay, we assessed bacterial growth by measuring the OD_600_ of the bacterial culture after incubation with each drug and comparing it to the growth of probiotics incubated without drugs (see Figure 5 and Supplementary Excel File S2). Our results demonstrated that all tested drugs exhibited a statistically significant growth-stimulatory effect on P1–P4 and P6, ranging from a 10% to 50% increase (*p* < 0.05) compared to control probiotics incubated without drugs. This observed effect could be attributed to the similarity in strains present in these probiotics. On the other hand, over 90% of the tested drugs significantly inhibited the growth of P7 and P8 by approximately 10% to 30%. Notably, cabergoline was the only drug that significantly increased the growth of P7 by 25%, with no discernible effect on P8. Aspirin had no impact on P7 but suppressed the growth of P8. The response of P5 to the tested drugs exhibited significant variation. Around 70% of the drugs inhibited its growth by 5% to 30%. Interestingly, cabergoline enhanced the growth of P5 by 25%, while other drugs such as Lisinopril, Digoxin, Diltiazem, Vildagliptin, and Norethindrone had no discernible effect on this probiotic. Our data are consistent with previous findings that report the effect of non-antibiotic drugs on the growth of gut microbes [50]. For example, loperamide was previously reported to decrease the growth of *B. longum* and increase the growth of *Lactococcus lactis* [51]. The observed probiotic-drug interactions might lead to unexpected effects not only affecting the probiotic strains but the entire gut microbial community. These interactions not only shape microbial composition but also represent a threat that might drive the development of antimicrobial resistance [52].

## 3. Discussion

The human gut is home to a diverse consortium of microbes, and this microbial composition plays a pivotal role in both health and disease [53,54]. Probiotics have gained attention as a supplement to promote a healthy gut, aiming to compensate for missing microbial species or functions [6]. However, the comprehensive impact of the administration of probiotics on gut composition and overall human health remains unexplored. In the current study, our objective was to assess the potential interactions of some commercially available probiotics, encompassing their immunomodulatory effects, antibacterial activities, antimicrobial resistance, and interactions with oral medications.

### 3.1. The Interactions between Probiotics, Host, and Microbes Are Evident

Previous reports indicated a potential risk and unexpected health hazards associated with the consumption of probiotics, particularly for at-risk populations such as immune-compromised or cancer patients [55,56]. Our data aligns with these reports as most of the tested probiotics were found to trigger the production of proinflammatory cytokines upon initial exposure. Previous reports showed that some strains of *Lacticaseibacillus* can trigger an initial immune response by inducing the secretion of IL-8 and IL-1β and generating reactive oxygen species [57]. The potential mechanism for this immunomodulatory activity of probiotic strains might be attributed to the production of active metabolites [58,59].

Furthermore, other reports emphasize that probiotics might exert antimicrobial activity against gut microbes, which can shape their composition and impact their function and human health. Previous reports support the notion that several species and strains from the *Lactobacillus genus* exert an antibacterial effect against major human commensal and pathogenic species, including *S. aureus* and *Clostridium difficile* [19,20]. Other reports showed that *B. infantis* exhibits antimicrobial activity against multiple intestinal pathogens [60] but the mechanism was mostly attributed to the production of acids or strong adhesion to the gut epithelia and hence the replacement of invading pathogens [60,61,62]. *L. lactis* is also known to produce antimicrobial compounds and antagonize pathogens by hindering their colonization [63,64]. The mechanisms underlying this antimicrobial activity involve the production of antimicrobial peptides and peroxides [65,66]. Other research showed that probiotics can restrict the colonization of pathogenic microbes [67]. This effect might be, in part, due to inducing the production of host β-defensin and immunoglobulin A, competition for nutrients, and the production of bioactive compounds that suppress or stimulate the growth of other microbes, as reviewed [68]. While this antimicrobial activity is desirable for the treatment of infectious diseases, it is important to consider the risks associated with this activity in shaping the gut microbial structure and function [69].

Our results are consistent with previous research, which reported the presence of *tetM* and *parC* in 20% and 76.5%, respectively, of 28 isolates from the *Lactobacillus genus* recovered from both environmental and human origins [70]. Moreover, there are multiple reports of species and strains from these genera containing AMR genes [71,72,73]. Although probiotics are not supposed to contain antimicrobial resistance (AMR) genes, many supplements on the market labeled as probiotics do not adhere to this guideline. Consequently, these supplements have the potential to escalate the AMR crisis, which was the leading cause of death in 2019 with around 1.27 million deaths and is projected to kill 10 M by 2050. Our findings align with several other studies that highlighted the potential role of probiotics in escalating the antibiotic resistance crisis [52] through the horizontal transfer of AMR genes. In a previous study, screening of 182 *Lactobacillus* species revealed multiple AMR genes conferring resistance to tetracycline and erythromycin in *L. amylotrophicus*, *L. ingluviei,* and *L. amylophilus* [74]. Interestingly, these genes were found to be flanked by mobilizing elements, suggesting a potential for horizontal gene transfer [74]. Moreover, the nucleotide sequence of the aminoglycoside resistance enzyme, *ant*(6), found in *L. animalis DSM 20602* and *L. amylophilus DSM 20533* showed high similarity to ant(6) in pathogenic bacteria such as *C. difficile*. This indicates the possibility of HGT between pathogenic bacteria and probiotic strains. Additionally, several probiotic strains have been reported to have AMR genes that are associated with phages or plasmids, indicating a possible mobilization of these genes via horizontal gene transfer [31]. These findings underscore the role of probiotics in the development of antibiotic resistance and the urgency to mitigate potential risks associated with their uncontrolled production and use.

Our data has revealed a broad range of probiotic-host-microbe interactions that could potentially pose a public health threat [75]. However, there were some limitations to our study. One challenge was tracing all observed activities to a single strain within each probiotic mixture. It is possible that some interactions were the result of the combined effect of multiple strains rather than the activity of a particular microbe. Additionally, we face difficulty achieving a 100% recovery rate for all strains in each probiotic, possibly due to antimicrobial activities between strains or suboptimal in vitro growth conditions. This limitation implies that the actual activity of probiotics might be greater than what we observed in our study. It is crucial to emphasize that our experimental results may not necessarily apply to the human body, particularly in the case of short-term probiotic use. This usage can result in transient gut microbiota changes without significant alterations in the microbial composition [76]. In addition, the viability of probiotics after oral digestion might differ from their viability under in vitro conditions with the use of a selective medium and the optimum growth medium. Previous reports suggest that most probiotics might undergo colonization resistance after oral administration due to rapid clearance from the gastrointestinal tract, inactivation by gastric secretions and enzymes, and competition with other microbes [77,78]. Thus, in vivo experiments are required to confirm the conclusion of this study.

### 3.2. Improved Regulations for the Use of Probiotics

In line with our data, which is consistent with previous reports, the general assumption that probiotics are safe is not accurate and does not reflect the holistic picture [79,80,81]. We believe that implementing improved regulations for probiotics is essential to minimize unexpected interactions and maximize the therapeutic outcome [82]. These regulations should include various aspects such as; (1) excluding probiotics from over-the-counter medications, (2) implementation of detailed safety studies focusing on antibiotic resistance genes [83], (3) comprehensive assessment of their interactions with other gut microbes including enhancement or suppression of their growth, (4) assessment of probiotics behavior with host microenvironment such as immune factors that might lead to selection pressure and development of virulence [84], (5) development of engineered strains for probiotic use with deleted or knocked out genes that do not conflict with the intended use such as antibiotic resistance genes [85], (6) detailed study of the product formulation and if there is any potential risk of provoking an immune response [86], and (7) inclusion of vulnerable population in probiotic studies such as patients with leaky guts, allergy, immune disorders, cancer, other critically-illness, and pregnancy [80]. Another crucial point to note is the accurate labeling of probiotics available on the market. The microbial strain that is labeled as probiotic should be validated by at least one double-blind, randomized, controlled clinical study demonstrating a statistically significant difference between the group that received probiotic supplementation and the placebo group [18]. Moreover, based on the standard definition, probiotic strains should not contain AMR genes, which is clearly not the case with most products that are commercially labeled as probiotics.

## 4. Materials and Methods

### 4.1. Microorganisms and Culture Conditions

We selected eight commercial probiotics for the current study and purchased them from local suppliers or through Amazon. The selection was based on the strains included in each probiotic and the intended use of the probiotic. For example, we excluded probiotics that have similar compositions and included probiotics that are intended for oral use only. A list of purchased probiotics, their microbial composition, and their intended use is shown in Supplementary Excel File S1. Culturing of probiotics was performed in four different media: De Man Rogosa and Sharpe (MRS), Tryptone Soy broth (TSB), Luria Bertani broth (LB), and Chopped Meat media (CMM). The selection of these media was based on prior information on the optimum growth media for the strains included in the probiotic mixtures. For example, MRS medium supports the growth of *Lactobacillus* strains, which were a main component in almost all tested probiotics. CMM medium supports the growth of *Bifidobacterium* TSB and LB support the growth of *Streptococcus* and other microbes. The crushed tablets or capsules of each probiotic were transferred to 5 mL of broth media, vortexed at 3200 for 10 s to ensure homogenous distribution of the powder in the broth, aliquoted as 200 μL into an empty 1.5 mL Eppendorf tube, and volume was completed to 1 mL by fresh medium and incubated at 37 °C under anaerobic and aerobic conditions for 24–48 h. Following incubation, we streaked each probiotic on an MRS and BHI agar plate, prepared a fresh broth inoculum, and aliquoted samples for long-term storage as a glycerol stock kept at −80 °C. We used a combination of morphological and biochemical methods to assess the recovery rate of distinct bacterial isolates from each probiotic mixture. These methods included gram staining, carbohydrate fermentation tests, and catalase tests, as previously described [87]. For the use of each probiotic mixture in subsequent studies, we developed and tested three protocols. First, culturing each strain recovered from each probiotic separately, then mixing in an equal ratio prior to usage in subsequent experiments; however, we thought that this method might not mimic the actual growth rate when the strains grow in a mixture. Second, making a combined glycerol stock of all the strains recovered from one probiotic and then culturing from this mixture for subsequent experiments Third, making a crude glycerol stock of the recovered mixture without separation of individual strains and using this stock to prepare fresh mixed cultures Overall, we did not notice a significant difference in the preliminary results; however, the third protocol was simpler and more realistic; therefore, we proceeded with all experiments with this protocol.

ATCC pure bacterial strains used in this study for the antimicrobial activity testing were ordered from Microbiologics (St Cloud, MN, USA), and revived according to the instruction protocol. Strains used in this study included: *L. planatrum* (ATCC 14917), *L. rhamnosus* (ATCC 7469), *L. casei* (ATCC 334), *S. aureus* (MRSA, BAA-2313), *S. bovis* (ATCC 33317), *S. salivarius* (ATCC 19258), *E. faecalis* (ATCC 29212), *Pseudomonas aeruginosa* (BAA-1744), *E. coli* (ATCC 33694), *C. albicans* (ATCC 18804), and *Saccharomyces cerevisiae* (ATCC 18824). Each microbe was cultured in the optimum growth medium and incubated at the optimum growth conditions.

### 4.2. Assessment of Pro/Anti-Inflammatory Activity of Probiotics

To assess the pro/anti-inflammatory activity of the probiotics, we tested them on the Caco-2 cell line (Cancer-Coli-2, HTB-37). These cells were originally isolated from colon tissue derived from a 72-year-old white male with colorectal carcinoma. We used TNF_α_ as an inducer of inflammation, followed by quantifications of IL-6, IL-8, and IL-1β cytokines in the cell supernatant using commercial ELISA kits. Briefly, Caco-2 cells were cultured in Dulbecco’s modified Eagle’s medium (DMEM) supplemented with 10% fetal bovine serum at 37 °C in an atmosphere of 5% CO_2_ (Eppendorf, Hamburg, Germany) until it reached 90% confluence. We used PBS buffer to wash cells multiple times to remove non-adherent cells and culture media. A Caco-2 monolayer was prepared using polycarbonate cell culture inserts (catalogue # 140654, Thermo Scientific, Waltham, MA, USA) covered with 100 μL of 1 mg/mL collagen Type 1 (catalogue # A1064401, ThermoFisher, Waltham, MA, USA) for 4 h then excess collagen solution was removed. We seeded Caco-2 cells on the top of the inset with a density of 0.5 × 10 [6] cells in 250 μL total volume of the medium suspension, and cells were left to attach overnight at 5% CO_2_, 37 °C, in a humidified incubator. We incubated the cells with each probiotic (1 µL at OD_600_ = 0.5) for 6 h followed by the addition of 1 μL of recombinant TNF_α._ (20 ng/mL, Sigma-Aldrich, St. Louis, MI, USA) in the case of stimulated cells, and then continued incubation for 18 h in total. In the case of non-stimulated cells, the incubation continued uninterrupted. Thereafter, we quantified the amount of IL-6 released using ELISA KITS (IL-6, catalog # BMS213HS, Invitrogen, Waltham, MA, USA), IL-8, catalog # BMS204-3, Invitrogen, and IL-1, β, catalog # KHC001, Invitrogen) according to the prescribed protocol. Negative control is non-treated cells with medium only (C1), while positive control is cells treated with TNF_α_ only (C2). Each analysis was repeated in triplicate.

### 4.3. Determination of Antimicrobial Activity of the Probiotics

To assess the activity of probiotics on representative human-associated yeast and bacteria, including both commensal and pathogenetic strains, we conducted an agar-well diffusion assay according to a previously published protocol with minor modifications [88]. Each microbe was cultured and maintained in the standard medium and growth conditions. 50 μL of each indicator strain (OD_600_ = 0.5) was applied to the solid agar plate (MRS, MHA, or PDA), streaked multiple times, and incubated for 2 h aerobically or anaerobically based on the optimum growth of each indicator strain. Following, we created holes in the agar and applied 100 μL of each probiotic culture (as a crude mixture) at 0D_600_ = 0.5 and incubated for 24 h anaerobically at 37 °C. Thereafter, we measured the diameter of the inhibition zone. Negative control is media only, while positive control includes the use of commercial antibiotics (10 µL of 1–5 μg/mL). The used antibiotics include amoxycillin, gentamycin, vancomycin, and chloramphenicol. The entire experiment is repeated in triplicate.

### 4.4. DNA Extraction, and Polymerase Chain Reaction Amplification

To determine the identity of the active bacterial isolates or to amplify antibiotic resistance genes, we performed DNA extraction, followed by PCR amplification using gene-specific primers, and finally sequencing (Nescience Company, Hong Kong, China). DNA was extracted as per manufacturer instructions (E.Z.N.A. Bacterial DNA Kit by Omega BIO-TEK). To identify the adequacy of the DNA extracts for the PCR-based assays, amplification of the 16S rRNA region was performed by PCR using two primers used in 16S rRNA sequencing: the 27 forward primer (AGA GTT TGA RSM TGG CTC AG) and the 1492 reverse primer (CGG TTA CCT TGT TAC GAC TT). For amplification of AMR genes, we used 7 different pairs of primers, as detailed in Supplementary Excel File S1.

The target antibiotic resistance genes have been selected based on computational prediction and antibiotic susceptibility tests. These genes confer resistance to (1) tetracyclines such as *tet*(M) and *tet*(L); (2) fluoroquinolone resistance gene *par (C)*; (3) aminoglycoside resistance gene fluoroquinolone acetylating aminoglycoside-(6)-N-acetyltransferase (aac 6-Ib-cr); (4) chloramphenicol resistance gene acetyltransferase (cat); and (5) erythromycin resistance genes *erm*(X) and *msr*(A). Each 50 μL reaction consisted of 40 μL of the ready-made PCR master mix, 5 μL primer mix, and 5 μL DNA (10–50 ng). The PCR master mix contained Taq DNA polymerase (0.05 U/µL), reaction buffer, 4 mM MgCl_2_, 0.4 mM of each dNTP, and Nuclease-free water. The reaction tubes were then placed in the PCR machine for 16S rRNA amplification. The detailed PCR reaction for 16s rRNA PCR amplification was as follows: 4 min, 94 °C, 1 cycle (amplification), 1 min, 94 °C, 23 cycles (melting). 30 s, 48 °C, 23 cycles (Annealing) and 2 min, 72 °C, 23 cycles (Extension for full length). The BLAST and NCBI databases were used to match the sequences to available sequences.

### 4.5. Assessing Antibiotic Resistance in Probiotics

To test the antibiotic resistance of the probiotics, we conducted three analyses:

Antibiotic susceptibility test using pre-loaded discs of penicillin G, amoxycillin, streptomycin, kanamycin, and ofloxacin Probiotics were applied as 100 μL of 24 h culture at OD_600_ = 0.5, and then the antibiotic discs were dispensed. The plates were then incubated at 37 °C for 24–48 h anaerobically, followed by measuring the inhibition zone. All experiments were repeated in three independent replicates.

Computational screening based on BLAST searches of possible resistance gene sequences (queried from the literature) on the NCBI database The original sequences were queried based on known antibiotic resistance genes in the main strains in commercial probiotics, which include *Lactobacillus acidophilus, Lacticaseibacillus rhamnosus*, *Lactobacillus gasseri*, *Ligilactobacillus salivarius*, *Lacticaseibacillus casei*, *Lacticaseibacillus paracasei*, *Lactobacillus delbrueckii*, and *Bacillus subtilis.* The aligned sequences were downloaded in FASTA format. Using MEGA11, a maximum likelihood phylogenetic tree was developed to illustrate the spread of the resistance enzyme among the species.

Whole genome screening of the Comprehensive Antibiotic Resistance Database available from McMaster University (CARD, https://card.mcmaster.ca/analyze/rgi, accessed on 20 September 2022) following the Resistance Genome Identifier (RGI) function The analysis was set to include perfect (P), strict (S), and loose (L) algorithms and reviewed the AMR genes of ≥40% matching identity. This selection helped to identify perfect matches in the query genome to existing AMR genes, mutations or unspecified variants to AMR genes, or possible arising AMR gene threats (Supplementary Excel File S2). This prediction is followed by wet lab validation, in which we synthesized primers for selected antibiotic resistance genes detected, followed by PCR amplification and product size matching.

### 4.6. Probiotics/Drugs Co-Incubation Assay

To investigate the effect of orally administered drugs on probiotics, we designed a co-incubation experiment in a 96-well plate format. We seeded the 96-well plate with 24 h of bacterial culture (a crude mixture recovered from the probiotics) that was centrifuged at 6000 rpm for 5 min (Sprout Mini Centrifuge, Heathrow Scientific, Vernon Hills, IL, USA), washed in double distilled water, recentrifuged, and resuspended in 1/5 strength TSB medium to an OD_600_ of 0.5, and incubated anaerobically for 24 h at 37 °C. Afterward, we added 150 of each drug (10 μmolar final concentration) to each specified well. We generated 160 unique reactions from the co-incubation of 8 probiotics and 20 drugs. The drugs have been selected to represent widely used oral medications such as painkillers, type 2 diabetes medications, antihypertensive drugs, and others. Another criterion we considered while selecting these drugs was the variation of chemical scaffolds, which increase the chance of microbial uptake or degradation. Each reaction was repeated in duplicate. The control reactions included media only (to exclude the effect of media components on the growth of probiotics), probiotics only (to measure the survival of probiotics without drugs to avoid false positive data from slow or fast-growing microbes), and drug only reactions. We measured OD_600_ before and after incubation using a microplate reader. Drugs used in this study included lisinopril, digoxin, aspirin, famciclovir, and ritonavir. diltiazem, carbamazepine, rivaroxaban, norethindrone, and vildagliptin. warfarin, amlodipine, carbimazole, and acarbose. loperamide, cabergoline, meloxicam, loratadine, and glimepiride. All drugs were purchased from Sigma Aldrich or reconstituted from their original dosage forms.

### 4.7. Statistical Analysis

We used GraphPad Prism V9 (GraphPad Software, La Jolla, CA, USA) to analyze all the data and generate graphs. Data are presented as mean ± standard deviation (SD). * indicates that results are significantly different at *p* < 0.05.

## 5. Conclusions

Our findings suggest that at least one probiotic tested induces mild inflammation in Caco-2 cell lines upon initial exposure, possesses antibiotic resistance genes that are transferable, and exhibits significant growth inhibitory activity against at least one microbial strain. Multiple orally administered drugs influence the survival of these probiotics, which might affect their colonization and efficacy. In summary, while probiotics have proven effective in the treatment of many diseases, it is essential to update and enhance their controlled testing, adverse interactions, therapeutic outcomes, approval, and administration [89]. Improved regulations for probiotics will provide clearer guidelines for their production, labeling, and administration, which will promote their safe and effective use.

## Data Availability

All generated data are included and available in the manuscript text or supplementary files.

## Supplementary Materials

The supporting information can be downloaded at: https://www.mdpi.com/article/10.3390/ijms241813783/s1.
